# Forensic Microbiological Analysis of Soil and the Physical Evidence Buried in Soil Obtained from Several Towns in Istanbul

**DOI:** 10.7759/cureus.22329

**Published:** 2022-02-17

**Authors:** Fatma Gül Efeoğlu, Hüseyin Çakan, Umut Kara, Taner Daş

**Affiliations:** 1 Institute of Forensic Sciences and Legal Medicine, Istanbul University-Cerrahpaşa, Istanbul, TUR; 2 Department of Biology and Microbiology, Çanakkale Onsekiz Mart University, Faculty of Arts and Sciences, Çanakkale, TUR; 3 Morgue Department, Histopathology Unit, The Council of Forensic Medicine, Istanbul, TUR

**Keywords:** microbial analysis, forensic soil microbiology, microbial identification, physical evidence, soil, forensic science

## Abstract

Background

The identification of bacterial species in the soil can be used for the differentiation of soil samples and physical evidence. This study aims to evaluate the importance of identifying microorganisms in the soil for forensic sciences. The study covered 20 regions identified and marked outside the settlement areas within the boundaries of Istanbul.

Methodology

Big and wide soil and forest areas were preferred. Four types of physical evidence samples were collected from the identified areas at the end of the first, second, and third months and then analyzed. The collected samples were physically embedded in the soil. In this study, 10 g of soil sample and four pieces of physical evidence (fabric, rubber, metal, and wood), sized 5 × 5 cm and buried 20-30 cm deep in the soil, contaminated with soil were collected for analysis and stored in sterile conditions. The microbiological identification analyses were conducted at the end of the predefined period and in the predefined order using first phenotypic (e.g., microscopic and macroscopic), followed by culture methods using advanced diagnostic analyses, such as API and matrix-assisted laser desorption/ionization-time of flight mass spectrometry.

Results

In the soil samples and the physical evidence samples collected, 83% bacteria and 17% fungus were identified. A database was set up for the study findings.

Conclusions

The presence of microorganisms in the soil and physical evidence samples contaminated with soil, which is crucial in the evaluation of criminal cases, was determined using microbiological analysis.

## Introduction

Crime scene investigation and the evidence obtained during this process are very important in solving judicial cases [1]. One of the physical pieces of evidence that enables us to establish a relationship between the crime scene-crime victim and the criminal is soil [2,3]. The evidential value of soil is due to its widespread availability at crime scenes and the transfer of it to the suspect. Soil or mud found on the suspect’s clothing, shoes, or car can be compared to the soil or mud at the crime scene [4]. Thus, it can be a finding that links the suspect or object to the crime scene. This finding can be used as evidence in forensic cases, such as murder, theft, smuggling, terrorism, traffic accident, and rape. Due to the complex structure, heterogeneity, and transferability of soil, clues about the origin and location of an unknown sample can be obtained [2].

Soil assumes different compositions and characteristic structures in different areas due to the influence of several natural factors, such as climate (humidity, temperature), basic material (main rock), relief (topography), living organisms, and time [2,4,5]. The versatile nature of microbial distribution in the soil gives us the chance to use microorganisms in identifying the soil. Based on findings from many ecological studies, microbial communities differ between various land uses and vegetation types [6]. In this study, our goal is to determine the contribution of the microbiological aspect of soil for forensic sciences using these differences. The most important result we expect is that the microorganisms in soil can be used as an indicator for the identification of objects contaminated with soil. Microbiologically, three groups of microorganisms make it possible to identify the soil and to specify the similarities and differences among different soil samples. Comparing the morphologies of fungal, bacterial, and actinomycetes colonies can help us identify soil samples.

In forensic microbiological analysis, several methods are used to examine the diversity and closely related microorganisms. However, none of these methods can be used as a definitive forensic application technique. No single approach is likely to be universally applicable. Thus, the error rates of microbial identification methods to be selected as forensic applications should be calculated, and their reproducibility should be measured in the laboratory [7,8]. Recently, matrix-assisted laser desorption ionization-time of flight mass spectrometry (MALDI-TOF MS) has emerged as a potential tool for microbial identification and diagnosis. Currently, this device is used in clinical applications. MALDI-TOF MS is fast, sensitive, high in discrimination, and economic [9]. It appears to be much more effective in terms of cost per sample and the time taken for identification compared to conventional and molecular identification methods [9]. MALDI-TOF MS can even successfully distinguish close species that could previously be separated by 16S rRNA gene sequences [10]. This technique has high sensitivity and specificity for identifying Gram-positive and Gram-negative bacteria and yeasts at the genus and species level [11]. In a study using a MALDI-TOF MS device with clinical filamentous fungal taxa, the species identification rate was higher when compared to conventional methods (conventional method: 80%, MALDI-TOF MS: 89%) [12]. In our study, we aimed to determine the most effective identification technique using both conventional methods and MALDI-TOF MS analysis.

## Materials and methods

Burial experiment: The field part

**Figure 1:**
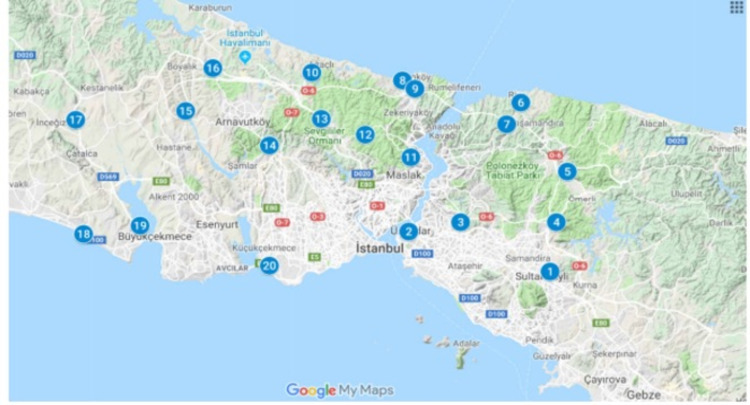
Field application map with numbers indicating sampling locations. Source and date: Google Maps, January 25, 2018.

**Figure 2:**
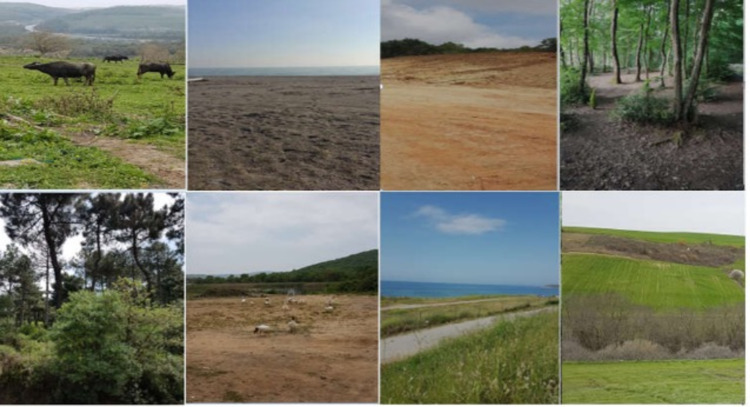
Examples of different field application regions.

Our research covered 20 regions identified and marked within the boundaries of Istanbul. Of these regions, 13 were selected from the continent of Europe (24%) and seven from Asia (29%) (Figure 1). Large soil and forested areas were preferred outside the settlements where evidence can be hidden (Figure 2). Location coordinates were obtained via a mobile phone with the Kocaman Version: 2.8 GPS application (Table 1). In our study, four pieces of physical evidence sized 5 × 5 cm (wood, fabric, rubber, and metal) served as the physical evidence samples. Each sample was wrapped in aluminum foil, sterilized in an autoclave at 121°C and 1 atm pressure for 15 minutes, and decontaminated. Each sample was buried at a depth of 20-30 cm, where the microbiological activity of the soil was the highest. They were kept separate from each other to avoid cross-contamination. The total burial area was designed not to exceed 1 m^2^. At the end of the first, second, and third months, four physical evidence samples and 10 g soil samples were collected from each region, placed in Petri dishes, and labeled.

**Table 1:** Detailed GPS locations of field applications areas.

Region number	Location	GPS coordinates
1	Aydos Forest - Kartal	40.937780, 29.226176
2	Fethi Paşa Grove - Üsküdar	41.031095, 29.028693
3	Kocatepe - Ümraniye	41.044383, 29.119472
4	Taşdelen - Çekmeköy	41.043976, 29.287036
5	Sarıpınar Hüseyin Roadside - Çekmeköy	41.116791, 29.306123
6	Riva - Beykoz	41.213194, 29.225026
7	Ali Bahadır Village - Beykoz	41.183203, 29.200827
8	Kilyos Coast Road - Sarıyer	41.244187, 29.018071
9	Kilyos Grave - Sarıyer	41.232427, 29.039647
10	Ağaçlı Road - Eyüp	41.255586, 28.859887
11	Hacıosman Grove - Sarıyer	41.136586, 29.032489
12	Belgrad Forest - Sarıyer	41.168742, 28.953722
13	Göktürk Natural Park - Eyüp	41.19006, 28.87646
14	Şamlar - Arnavutköy	41.152608, 28.786239
15	Dursunköy - Arnavutköy	41.201725, 28.638761
16	Tayakadın - Arnavutköy	41.261793, 28.687110
17	Çatalca	41.18901, 28.44627
18	Kumburgaz - Büyükçekmece	41.02722, 28.45972
19	Büyükçekmece Lake - Büyükçekmece	41.038703, 28.559337
20	Florya Forest - Bakırköy	40.98305, 28.78583

Culture and MALDI-TOF-MS analysis

For soil samples, 9 mL of 0.9% tryptic soy broth (bunion) and/or isotonic sodium chloride (physiological saline) was poured into a Petri dish on 1 g of soil and diluted at a ratio of 1-10. The Petri dish was then kept in an incubator at 37°C for one to two hours to activate the microorganisms and allow their passage from the soil into the liquid. For measuring pH, 5 g of soil from each of the samples was placed in the tubes. Subsequently, 10 mL of distilled water was added to the tubes. The mixture was then vortexed for 1 minute. Then, it was centrifuged at 5,000 revolutions per minute (rpm) for five minutes. The remaining liquid was poured into other tubes, and each sample was measured using a standardized digital pH meter. For physical evidence samples, 4.5-5 cc of 0.9% isotonic sodium chloride was dropped on the samples in Petri dishes. Thus, the samples were wetted by washing with physiological saline.

**Figure 3:**
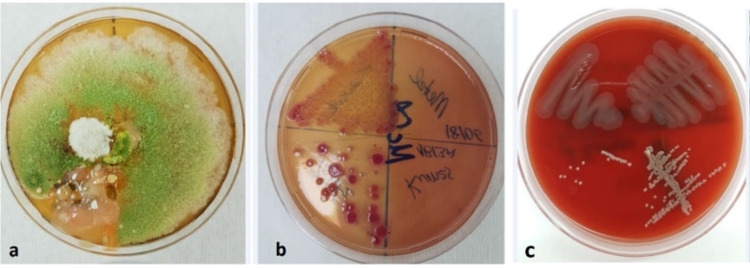
Colony images of microorganisms. (a) Region 4: malt extract agar medium. (b) Region 1: MacConkey agar medium. (c) Region 2: sheep blood Columbia agar medium.

**Figure 4:**
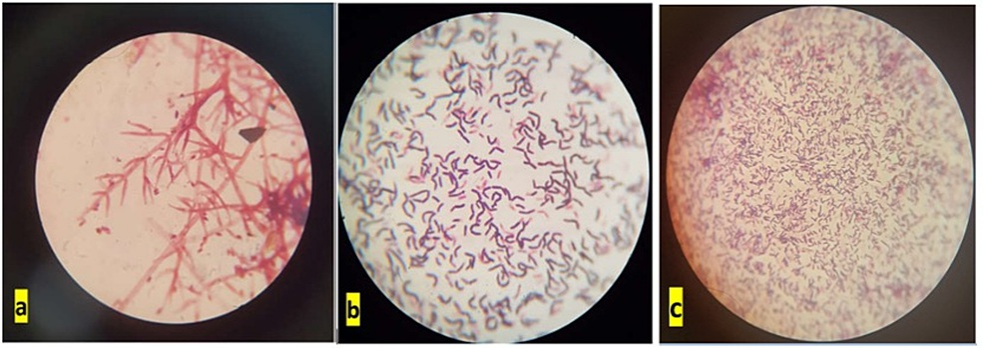
Images at ×10 and ×100 magnification. (a) Region 2 soil sample: *Alternaria* spp. and its conidia. (b) Region 2 metal sample: *Bacillus megaterium*. (c) Region 2 rubber sample: *Bacillus subtilis*/*Micrococcus* spp.

Swab samples were obtained using a sterile swab from each Petri dish to sample the entire area. The samples were then inoculated on tryptic soy agar (TSA), malt extract agar (MEA), and MacConkey agar (MAC) growth medium by applying the single colony streaking technique using a sterile needle loop and swab stick, with the Bunsen burner next to it. The samples inoculated in the TSA and MAC media were incubated at 37°C for 24-48 hours. The samples inoculated in the MEA medium were kept at room temperature at 22-24°C for 7-14 days. For culture examination of Petri dishes after incubation, the plates were evaluated and photographed macroscopically concerning color, odor, hemolysis, and shape (Figure 3). For microscopic examination, colonies were marked on the culture. They were subjected to the Gram stain procedure. After staining, microorganisms were examined under a binocular light microscope (Figure 4). KOH test was performed for colonies that could not be distinguished as positive or negative on Gram staining. In addition, we performed catalase test for colonies with suspected streptococci, coagulase test to determine *Staphylococcus aureus* species, oxidase test for identifying Gram-negative (*Enterobacteriaceae*) species, motility test to determine bacterial motility (flagella), API 20C for fungal identification, and API 20 Strep for identification of streptococci. MALDI-TOF MS (MALDI Biotyper®, Bruker Daltonic, Billerica, MA, USA) was used for samples that could not be typed as advanced identification analysis. For this analysis, 24-48-hour fresh and pure cultures were prepared in a 5% sheep blood Columbia agar medium. Subsequently, 0.005 g of α-cyano-4-hydroxycinnamic acid (HCCA) matrix (stored at 2-8°C) material was weighed on a microbalance and transferred to a tube. It was used as an organic solvent to dilute the HCCA matrix material. For preparing the organic solvent, 237.5 μL of water was added into a 1 mL Eppendorf tube. Then, 250 μL of acetonitrile and 12.5 μL of trifluoroacetic acid were added to the water. The solution was mixed by vortexing. Following this, 450 μL of this prepared solution was added to the HCCA matrix and vortexed. Samples from pure cultures were mixed with the matrix on a conductive metal plate. After crystallization of the matrix and microbial material, the metal plate was placed in the mass spectrometer. The 10 best-matching identification results were displayed on the computer. Identification results with score values above 2.0 were considered for the determination of the respective species (Table 2).

**Table 2:** Computer display of identification results of B7 coded 16th region wood sample in MALDI-TOF MS database: Arthrobacter gandavensis. (++) Secure genus identification, probable species identification; (+) probable species identification; (-) not reliable identification. MALDI-TOF MS: matrix-assisted laser desorption/ionization-time of flight mass spectrometry

Rank (Quality)	Matched pattern	Score value	NCBI identifier
1 (++)	*Arthrobacter gandavensis* DSM 15046T DSM	2.036	169960
2 (+)	*Arthrobacter koreensis* DSM 16760T DSM	1.922	199136
3 (-)	*Arthrobacter luteolus* DSM13067T DSM	1.416	98672
4 (-)	*Pseudomonas flavescens* DSM 12071T HAM	1.228	29435
5 (-)	*Arthrobacter oxydans* IMET 10684T HKJ	1.14	1671
6 (-)	*Sinomonas atrocyanea* HKI 10432 HKJ	1.132	37927
7 (-)	*Phoma exigua* ssp. exigua CBS 431_74 CBS	1.113	79605
8 (-)	*Arthrobacter ureafaciens* DSM 20126T DSM	1.113	37931
9 (-)	*Arthrobacter citreus* IMET 10680T HKJ	1.107	1670
10 (-)	*Thauera aromatica* K172 MPB	1.096	44139

Statistical analysis

Data analysis was done using SPSS Statistics version 20 (IBM Corp., Armonk, NY, USA). The significance value was taken as 0.05, and values below 0.05 were considered statistically significant. Fungi and bacteria were grouped and studied separately. For the normality of the distribution of both groups, Shapiro-Wilk test, Kruskal-Wallis H test, and analysis of variance (ANOVA) test for the differences between groups, Mann-Whitney U test for pairwise comparisons, and Levene’s tests for the homogeneity of group variances were performed. For ordinal data, arithmetic mean, standard deviation, minimum, and maximum were computed, and for nominal data, frequency and percentage were computed.

## Results

Identification

**Figure 5:**
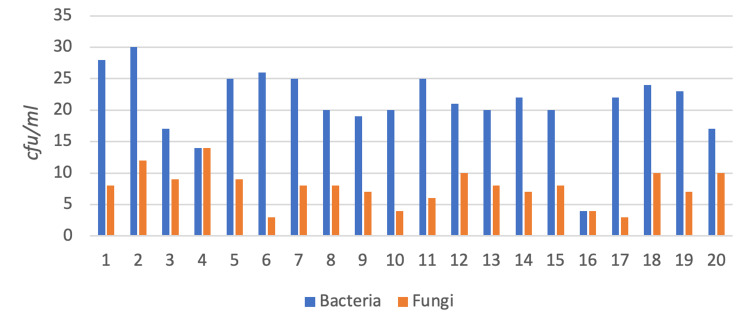
The distribution of microorganisms by region.

While evaluating the data, the data of the third month with the widest variety was taken as the basis. In case the physical samples in the burial areas were missing (e.g., due to theft and construction), data from another month were used. From the analyses of soil and physical samples, 577 microorganisms were typified. The distribution of these microorganisms by region is shown in Figure 5. Overall, 83% bacteria and 17% fungus were identified in the soil samples and physical evidence samples.

The highest rate of fungal species (30%) was detected in wood samples, and the highest rate of bacterial species (30%) was detected in rubber samples.

**Figure 6:**
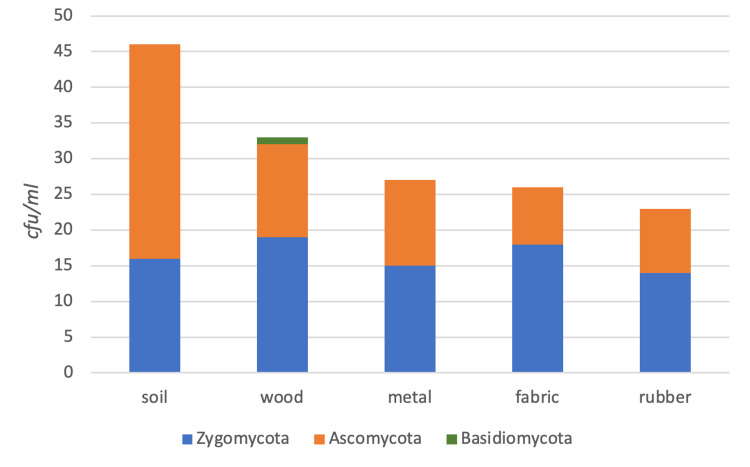
Fungal composition of the samples.

**Figure 7:**
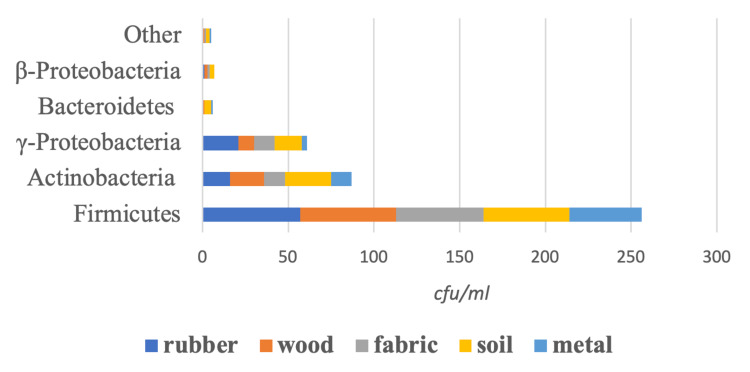
Bacterial composition of the samples.

The identified fungi included Zygomycota (53%), Ascomycota (46%), and Basidiomycota phylum (1%) (Figure 6). The identified bacteria included Firmicutes (61%), Actinobacteria (21%), γ-Proteobacteria (14%), β-Proteobacteria (2%), Bacteroidetes (2%), and other phyla (1%) (Figure 7).

pH

The mean ± standard deviation of pH measurements in 20 different regions was 6.98 ± 0.71. The minimum value was 5.1, and the maximum value was 7.70. To evaluate the effect of pH on bacterial diversity in 20 different regions, pH values were divided into three categories. pH values between 5 and 6 were included in category 1, values between 6 and 7 were included in category 2, and values between 7 and 8 were included in category 3. A plan was created that allowed us to see the difference in bacterial diversity according to categories. The Shapiro-Wilk test was used to test the normality of the data distribution in these three categories. It was determined that the data were not appropriate for normal distribution concerning groups (p < 0.05). Hence, the difference between the three groups was evaluated using the Kruskal-Wallis H test. According to the test results, a significant difference was found between pH levels in the three categories concerning bacterial diversity (p = 0.023). Pairwise comparisons were made using the Mann-Whitney U test to determine the pH range categories responsible for the difference. A significant difference was found between categories 1 and 2 (p = 0.028), as well as between categories 1 and 3 (p = 0.039). Thus, category 1 (pH range of 5-6) was thought to have a different bacterial diversity than other categories.

For fungi, the Shapiro-Wilk test was used to test the normality of the data distribution in the three categories. It was determined that the data were not appropriate for normal distribution in terms of groups. The data in the first group were not normally distributed (p < 0.05). Hence, the Kruskal-Wallis test was used to determine the difference between the three groups for fungi. Accordingly, no significant difference was found between the pH levels in the three categories regarding fungal diversity (p = 0.230).

Humidity and temperature

The mean ± standard deviation values of humidity for 20 different regions were 85,545 ± 5.17. The minimum value was 75.60, and the maximum value was 95.70. The mean ± standard deviation of temperature for 20 different regions was 19.445 ± 2.057. The minimum value was 12.60, and the maximum value was 22.30. The mean ± standard deviation of the maximum temperature was 24.5 ± 2.323. The mean ± standard deviation of the minimum temperature was 16.635 ± 1.341.

Physical evidence samples

Four different models of physical evidence samples were determined as the first group (wood), second group (fabric), third group (rubber), and fourth group (metal). The data distribution of bacterial species in the four different groups was normal distribution according to the Shapiro-Wilk test results (p > 0.05). Concerning homogeneity of variances in the four different groups, Levene’s test statistic was calculated and evaluated. Group variances were homogeneous (p > 0.05). It was examined whether there was a difference between the groups concerning the number of bacterial species. There was a statistically significant difference in the number of bacterial species in the four different groups according to the ANOVA test (p = 0.040).

Shapiro-Wilk test results showed that the fungal species data distribution in four different groups was not normal distribution (p < 0.05). Hence, the Kruskal-Wallis H test was performed, and no significant difference was found concerning fungal diversity in the four different groups (p = 0.474).

## Discussion

A wide variety of physical and chemical factors, such as pH, nutrient levels, quantity and quality of organic carbon, moisture, and oxygen levels, can have a major impact on microbial communities in near-surface and deepest horizons [13-16]. The fact that soil pH is the most important driver of bacterial community composition has been confirmed in many studies, especially those focusing on forest soils [17-19]. In the statistical evaluation to determine the effects of pH on bacterial diversity, it was observed that the pH range of 5-6 had a different bacterial diversity than other pH ranges. It has been confirmed by our study that bacteria need narrow pH ranges for optimal growth, whereas fungi generally require wider pH ranges for optimum growth.

In addition to physicochemical parameters in soil differentiation, the type and presence of plants are among the determining factors for many soil microbial communities [20,21]. Certain profile features are specific to particular ecosystems. These features facilitate the identification of ecologically diverse areas and can allow character profiles to be determined. Thus, it can be determined that the sample collected from a suspect originated from a field, not from a forest [22]. In our study, plant-specific bacterial species were detected, such as *Paenibacillus macerans* and *Rhizobium radiobacter*. In addition, the abundance and functional importance of Actinobacteria and Proteobacteria phyla on forest soils have been reported by previous studies [23]. Our findings showed the presence of Actinobacteria phylum as *Actinomyces* spp., *Micrococcus luteus*, *Streptomyces* spp., and *Arthrobacter* spp., and the presence of Proteobacteria phylum as *Pseudomonas aeruginosa*, *Acinetobacter* spp., *Proteus mirabilis*, and *Aeromonas* spp. in forest lands.

*Bacillus* species, which was the most common type of bacteria (47%) in our study, is a soil-borne spore and can be easily carried everywhere by the wind. *Bacillus* species may produce bacteriocins, which can inhibit the growth of other microorganisms or even cause their death. *Bacillus *spp. display antibacterial and antifungal activity against different plant pathogens by producing components such as peptides, lipopeptides, phospholipids, and polyenes [24]. In our study, it was observed that the bacterial diversity (69%) in the soil was higher than the fungal diversity (31%). The reason could be the inhibitory effect of *Bacillus *species.

It has been stated that fungal communities can be a stronger target for forensic soil investigations than bacterial communities [6,25]. Each fungal species has specific nutritional and habitat requirements [26]. Different crime scene investigations have shown that fungal communities are valuable forensic evidence that can distinguish distinctive regions despite the proximity of the location [6,26]. The closest distance between the regions in our field study was 3.17 km between the eighth and ninth regions, and the longest distance was 72.8 km between the fifth and seventeenth regions. In our study, *Penicillium roqueforti*, *Penicillium digitatum*, *Beauveria bassiana*, *Mucor circinelloides*, *Rhizopus microsporus*, and *Cladosporium* spp. fungal species were identified in different regions. Thus, the question “was the crime committed in that place?” was answered with this power of discrimination.

Four different physical evidence models

It has been stated that *Bacillus megaterium *and *Pseudomonas *spp. reduce the corrosion of stainless steel in inorganic medium and increase the corrosion in organic medium. Metal physical evidence samples contaminated with soil were made of stainless steel and we detected *Bacillus megaterium* and *Pseudomonas* species on them. Fungi produce organic acids due to their metabolism and lower the pH of the environment. Thus, they play a role in metal corrosion [27]. In our study, 42% *Mucor* spp. was detected on metal physical evidence samples.

In recent years, many bacterial species have been shown to use carbon and rubber as the sole source of energy. The strongest Actinomycete representatives of this group are *Actinoplanes*, *Streptomyces*, and *Micromonospora *species. On our rubber model physical evidence samples, *Streptomyces *spp. and *Micrococcus* spp. were detected. In studies investigating the degradation of rubber hydrocarbon by fungi, *Penicillium* spp., *Aspergillus *spp., *Fusarium solani*, and *Cladosporium cladosporioides* were shown to play a role [28]. In our study, *Aspergillus *spp., *Aspergillus nidulans*, *Aspergillus niger*, *Cladosporium *spp., *Penicillium chrysogenum*, and *Penicillium discolor* were detected in the rubber physical evidence samples.

In most terrestrial environments, fungal degradation of wood is predominant [29]. A higher rate (30%) of fungal species diversity was detected in our wooden physical evidence samples than others.

Bacterial degradation of textile materials is more intense than fungal degradation. *Bacillus subtilis* and *Pseudomonas* are found in raw cotton [30]. *Bacillus subtilis* was also detected in the physical evidence of the fabric (cotton) we used in our study. In the study on the biodegradation of natural polymers, *Mucor* spp. was identified to cause degradation in textile materials, especially cotton fibers in the soil (105). In the fabric (cotton) physical evidence samples, the fungus species detected at the highest rate (50%) was *Mucor *spp.

The physical models we used in our study represent evidence, such as a knife (metal), found at the crime scene, shoes (rubber), and clothing (fabric) worn by the suspect at the crime scene. By identifying microorganisms based on this evidence, the soil-bacteria profile in the area where a victim’s corpse was found can be matched with the soil-bacteria profile on the suspect’s physical tools. Consequently, the diversity of microorganisms found in soil and soil-contaminated materials increases the evidence value of soil in criminal investigations.

Limitations

Our retrospective and auto-controlled study with model physical evidence of soil contamination was conducted in the spring season when microbial activity and diversity were the highest; however, the effects of seasons on soil microbial communities could not be detected. Therefore, this aspect should be considered in the interpretation of our results. The summer and winter seasons will likely cause changes in microbial communities due to carbon and nitrogen amounts, cyclical changes in tree physiology, and changes in soil moisture and temperature. Our study, carried out only in the spring season, does not include cyclical changes in the summer and winter seasons when physicochemical changes occur.

## Conclusions

Physical evidence (e.g., clothes and tools) contaminated with soil, which has considerable significance as proof in the investigation of criminal cases, was useful for forensic sciences by way of microbiological analysis. The findings suggest that microorganisms in the soil can be used as indicators for soil identification. This study, which was a preliminary work to solve a judicial case, has proven that soil-contaminated physical evidence can be used in solving crimes/incidents that have occurred in Istanbul. The data collected for Istanbul were converted into a model that can be used in future studies.
